# Virtual-Augmented Reality and Life-Like Neurosurgical Simulator for Training: First Evaluation of a Hands-On Experience for Residents

**DOI:** 10.3389/fsurg.2022.862948

**Published:** 2022-05-19

**Authors:** Salvatore Petrone, Fabio Cofano, Federico Nicolosi, Giannantonio Spena, Marco Moschino, Giuseppe Di Perna, Andrea Lavorato, Michele Maria Lanotte, Diego Garbossa

**Affiliations:** ^1^Department of Neuroscience “Rita Levi Montalcini”—Unit of Neurosurgery, University of Turin, Turin, Italy; ^2^Humanitas Gradenigo, Turin, Italy; ^3^Dipartimento di Medicina e Chirurgia - Neurochirurgia, Università degli Studi di Milano Bicocca, Milan, Italy; ^4^Fondazione Istituto di Ricovero e Cura a Carattere Scientifico (IRCCS) Policlinico San Matteo, Pavia, Italy; ^5^UpSurgeOn S.r.l., Assago, Italy

**Keywords:** simulator, training, virtual reality, life-like actuation, brain, neurosurgery, residents

## Abstract

**Background:**

In the recent years, growing interest in simulation-based surgical education has led to various practical alternatives for medical training. More recently, courses based on virtual reality (VR) and three-dimensional (3D)-printed models are available. In this paper, a hybrid (virtual and physical) neurosurgical simulator has been validated, equipped with augmented reality (AR) capabilities that can be used repeatedly to increase familiarity and improve the technical skills in human brain anatomy and neurosurgical approaches.

**Methods:**

The neurosurgical simulator used in this study (UpSurgeOn Box, UpSurgeOn Srl, Assago, Milan) combines a virtual component and a physical component with an intermediate step to provide a hybrid solution. A first reported and evaluated practical experience on the anatomical 3D-printed model has been conducted with a total of 30 residents in neurosurgery. The residents had the possibility to choose a specific approach, focus on the correct patient positioning, and go over the chosen approach step-by-step, interacting with the model through AR application. Next, each practical surgical step on the 3D model was timed and qualitatively evaluated by 3 senior neurosurgeons. Quality and usability-grade surveys were filled out by participants.

**Results:**

More than 89% of the residents assessed that the application and the AR simulator were very helpful in improving the orientation skills during neurosurgical approaches. Indeed, 89.3% of participants found brain and skull anatomy highly realistic during their tasks. Moreover, workshop exercises were considered useful in increasing the competency and technical skills required in the operating room by 85.8 and 84.7% of residents, respectively. Data collected confirmed that the anatomical model and its application were intuitive, well-integrated, and easy to use.

**Conclusion:**

The hybrid AR and 3D-printed neurosurgical simulator could be a valid tool for neurosurgical training, capable of enhancing personal technical skills and competence. In addition, it could be easy to imagine how patient safety would increase and healthcare costs would be reduced, even if more studies are needed to investigate these aspects. The integration of simulators for training in neurosurgery as preparatory steps for the operating room should be recommended and further investigated given their huge potential.

## Introduction

In recent years, growing interest in simulation-based surgical education has led to various practical alternatives for medical training (1). Factors such as the cost-effectiveness of the operating room, medico-legal and ethics implications, the actual restrictions for residents in terms of hours spent on work-related activities, and reduced time availability for surgical instruction during surgical activities are the elements most likely connected with a reduction in operative case volume during residency (2). After recent changes in disease management and technological advances, especially for surgical subspecializations, a growing attention has been paid to patient safety during surgical care, keeping back residents from the operating rooms while redirecting them into more administrative mansions (3).

On the other hand, up to 50,000 additional neurosurgeons are estimated worldwide to face the critically growing needs in surgical care (4). A net discrepancy between the need for new experienced neurosurgeons and their actual surgical experience is coming to light over time.

Traditionally, practical solutions for surgical training implementation include cadaveric and animal models and abroad fellowships (2, 3). However, some drawbacks and limitations must be taken into account. Neurosurgical anatomy is highly specific for human brain and does not compare well with animal specimens. Moreover, the management of cadaveric models entails ethical concerns and high maintenance costs, as well as specific and highly equipped structures for their preservation. In all these cases, practical training on these models allows only a partial anatomical practice, excluding the pathologic aspects of neurosurgical experience.

Nowadays, international training for surgical residents is considered desirable for a complete neurosurgical education in this particular medical field (5). Unfortunately, after the COVID-19 pandemic outbreak, surgical activities and international exchange programs have been dramatically reduced (6–8).

To counter the lack of surgical exposure, more recently, courses based on virtual reality (VR) and three-dimensional (3D)-printed models are available (2, 3). In particular, we validated a hybrid (virtual and physical) neurosurgical simulator equipped with augmented reality (AR) capabilities that can be used repeatedly to increase familiarity and improve technical skills in human brain anatomy and neurosurgical approaches.

## Materials and Methods

The neurosurgical simulator used in this study (UpSurgeOn Box, UpSurgeOn Srl, Assago, Milan) combines a virtual and a physical component with an intermediate step to provide a hybrid solution. The virtual part is based on an application which allows for the interactive exploration of 3D anatomical models and animations, both in a purely virtual environment and in an AR projection of the physical simulator (hybrid). These tools are designed to be integrated into a 3-step training sequence: mental training (based on a virtual environment), hybrid training (based on virtual models projected onto the physical simulator through a mobile device), and manual training (based on the physical simulator).

### Virtual Reality

Through the “Neurosurgery” mobile application, it is possible to explore the different surgical approaches using a smartphone or tablet.

A total of 9 surgical approaches (pterional, mini pterional, frontal monolateral, supraorbital, temporal, mini temporal, retrosigmoid, mini retrosigmoid, interhemispheric, and suboccipital) and 2 pathological modules for brain aneurysms and pituitary adenomas are available. Each virtual exploration uses 3D anatomical models and animations to support a Mental Training system aimed at understanding a patient's positioning according to a specific craniotomy and a specific intradural target.

**Figure 1:**
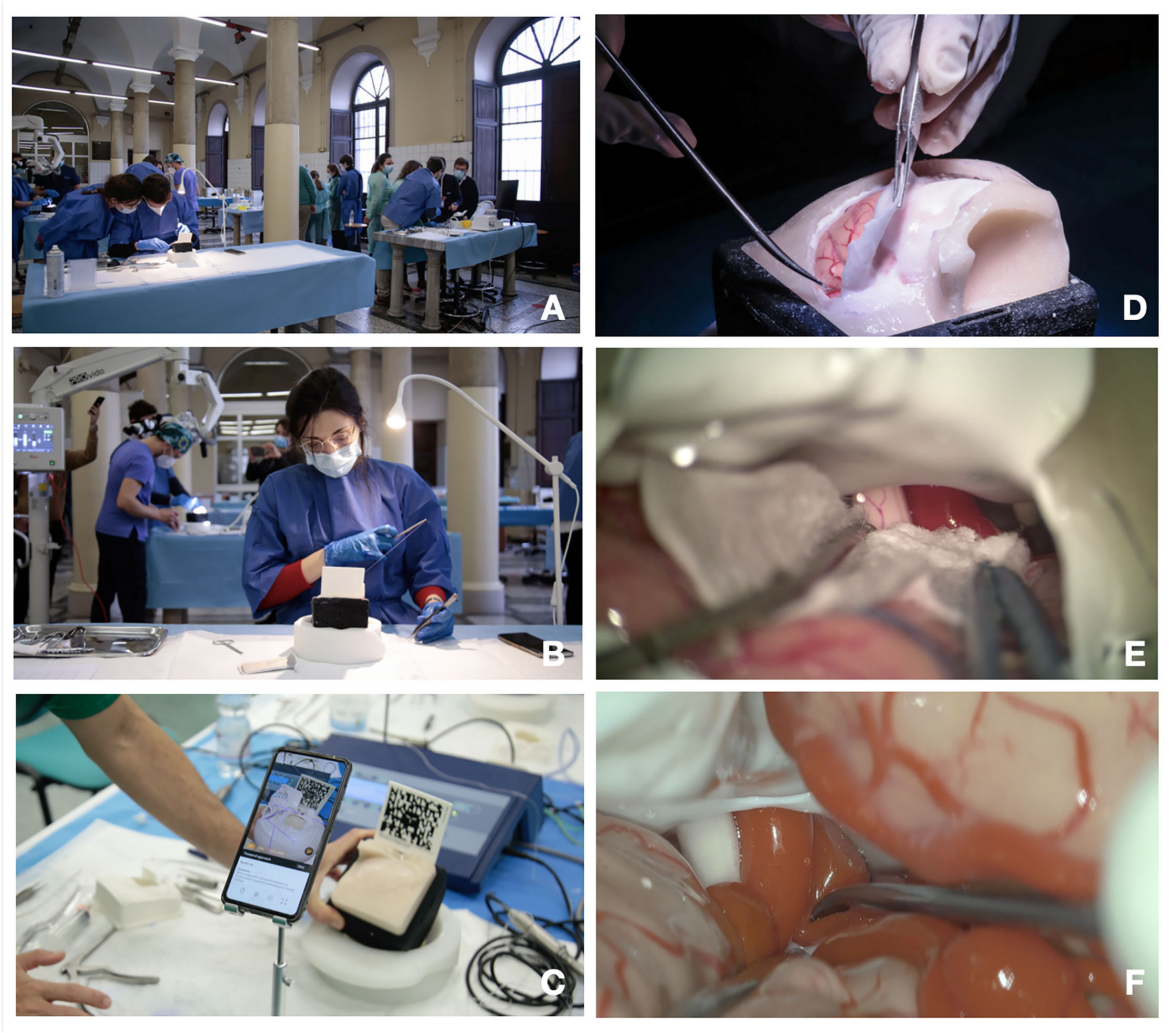
**(A–E)** Training steps. **(A)** Training laboratory set up; **(B)** suturing exercise; **(C)** augmented reality (AR) for craniotomy and approach planning; **(D)** dura opening on the pterional approach simulator box; **(C)** microscopic intradural phase through pterional approach with gentle brain retraction; **(E,F)** microscopic exploration and dissection through pterional approach of the carotid artery, optic nerve, and sylvian vessels.

The study of each approach in 3D mode is divided into 6 phases (Figures 1A–D) as follows:

1. Craniotomy selection: One can select among pterional, mini pterional, frontal monolateral, supraorbital, temporal, mini temporal, retrosigmoid, mini retrosigmoid, interhemispheric, and suboccipital approach.
2. Target selection: One can select specific anatomical (non-pathological) structures visible from the craniotomy previously selected.
3. Patient positioning: An artificial intelligence (AI)-based system calculates in real time a range of correct patient's body/head positionings according to the craniotomy and the target selected.
4. Surgical approach: This step involves an interactive 3D animation of the surgical steps of the approach.
5. Microsurgical exploration: This step simulates the microscope view using the gyroscopic technology of the hardware. Furthermore, tissues can be deformed by tapping the screen to expose the deep anatomy.
6. Closure: An interactive step that explains, through an animation, the reconstruction technique.

### Augmented Reality

**Figure 2:**
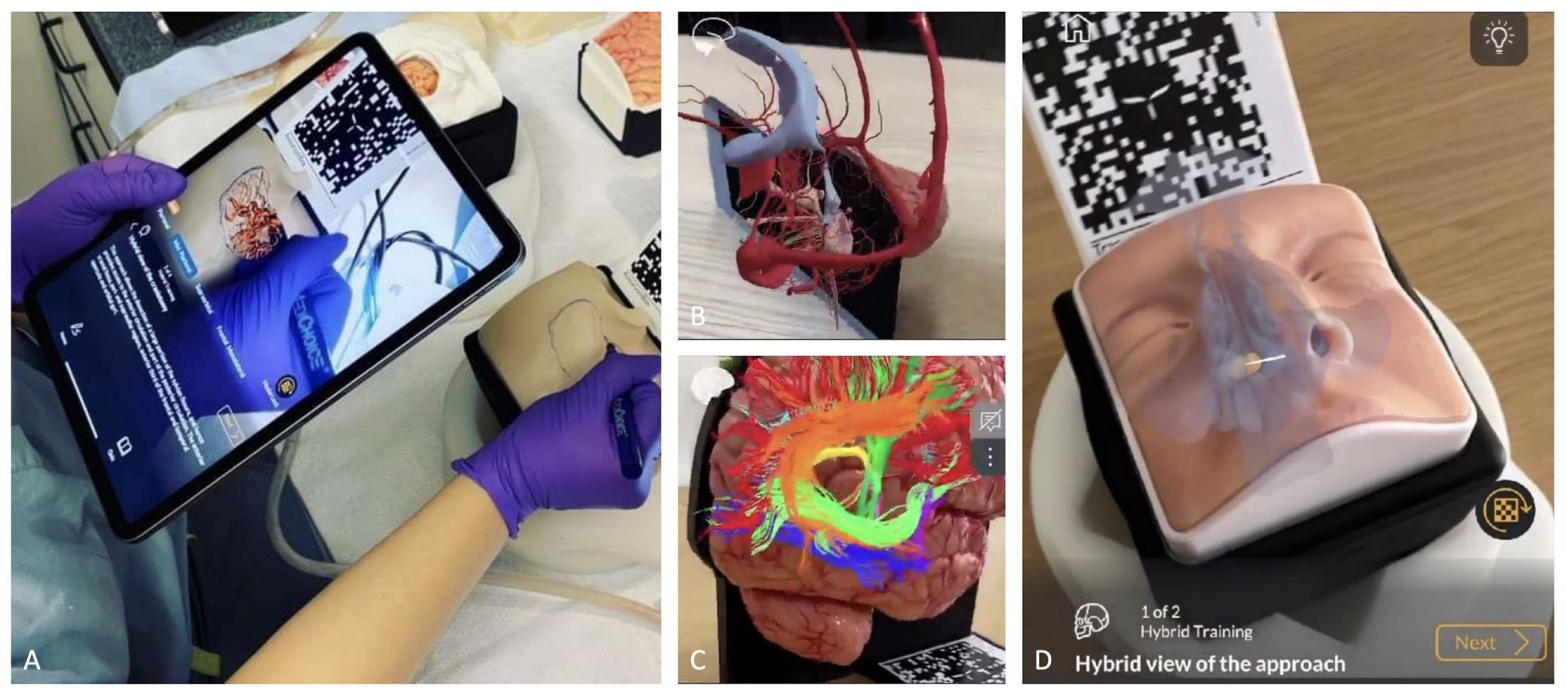
**(A-D)** The role of AR and the hybrid simulation. **(A)** AR of a pterional approach. Before performing the craniotomy, it is possible to see the vessels and the brain under the skull by framing the specific QR code through tablet and/or smartphone app; **(B)** brain parenchyma has been hidden to allow better evaluation of dural venous system and ventricular system; **(C)** AR used to better evaluate white matter fiber anatomy; **(D)** application of AR in endoscopic endonasal approach allows to identify anatomical landmarks before entering the nose.

The application software presents a module dedicated to the use of AR able to project 3D anatomical models and animations on the physical simulator, acting as a guide to plan the hands-on surgical approach (Figure 2).

This phase, defined by the term “Hybrid Training,” includes:

1. Patient positioning in AR: using AR it is possible to view the patient's entire skull on the simulator and modify the position of the head on the three axis. The AR system also allows to view the position of the target on the screen, before visualizing it on the physical simulator.
2. Craniotomy planning in AR: the Application projects on the physical skull several possible craniotomies.
3. Surgical steps in AR: the software reproduces the surgical approach step by step to understand which tasks will be carried out on the physical model.

### Physical Simulator

**Figure 3:**
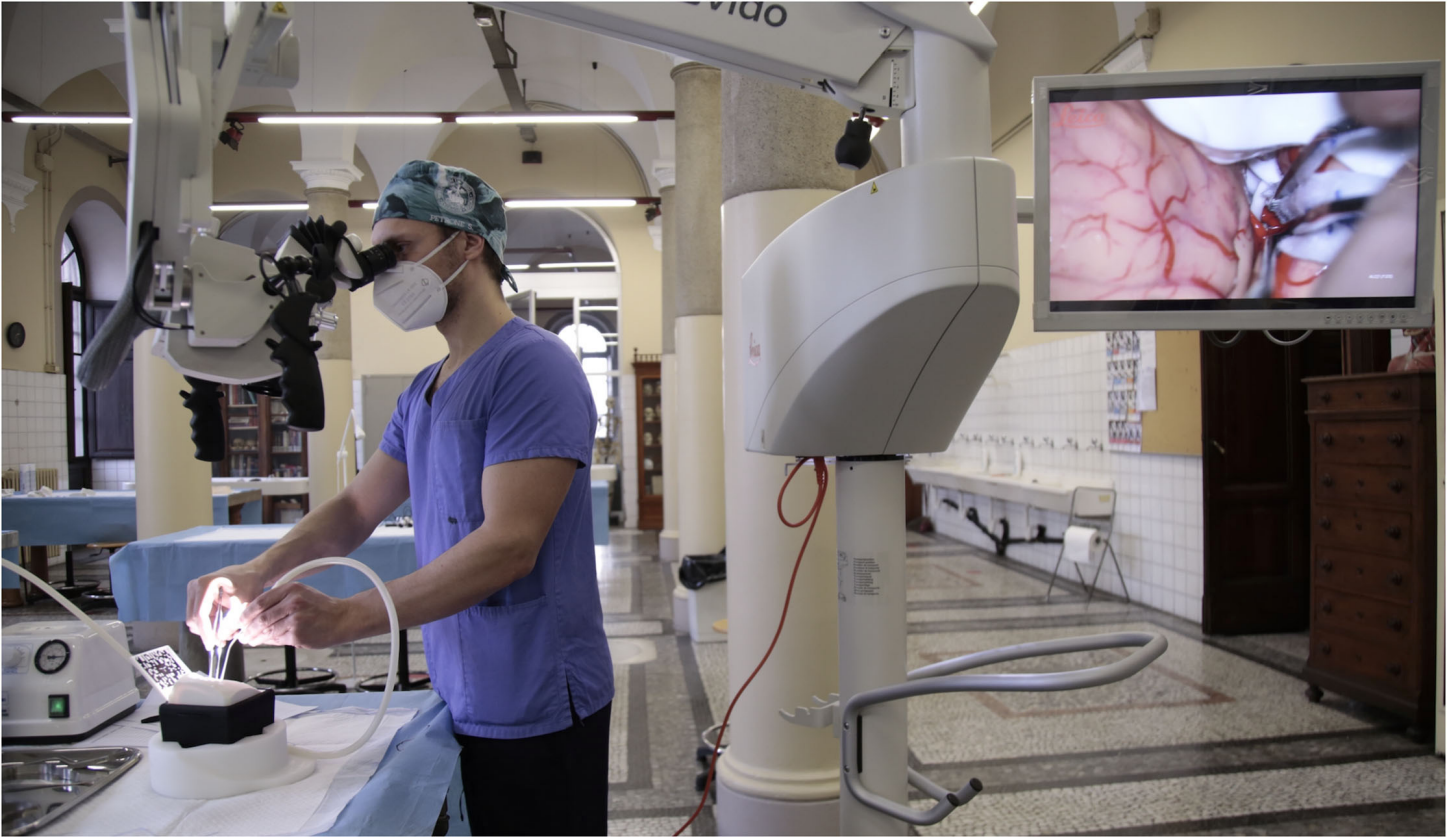
Workstation set up with microscope and simulator.

The simulator is designed to reproduce different surgical approaches (pterional, temporal, retrosigmoid, interhemispheric, suboccipital, and transsphenoidal) and different patient positions *via* a semi-spheric support. Through interchangeable skulls, one can perform multiple craniotomies and dural openings using the same deep microanatomical scenario, which can be explored under the microscope/exoscope/endoscope once the skull and dura are opened (Figures 1E,F, 3).

Once the craniotomy has been performed, it is then possible to replace the skull and start again.

### Data Collection

The first reported and evaluated practical experience on the anatomical 3D-printed model was conducted at the Department of Neuroscience “Rita Levi Montalcini” in Turin, Italy.

A total of 30 neurosurgery residents were involved. The first phase of the workshop consisted in using the AR application. The residents had the possibility to choose a specific approach (pterional, subtemporal, retrosigmoid), to focus on the correct patient positioning, and to go over the chosen approach step-by-step by interacting with the model through AR application.

Next, each practical surgical step on the 3D model was timed and qualitatively evaluated by 3 senior neurosurgeons. The training comprehended 4 standardized surgical moments: craniotomy, opening of the dura mater, reaching the pre-assigned target, and closing of the dura mater. Surgical loupes and a surgical microscope were available. For the pterional approach, optic nerve, middle cerebral artery (MCA), and III cranial nerve were identified as the principal targets of interest. Similarly, the IV cranial nerve and posterior cerebral artery (PCA) were chosen for the subtemporal approach, while VII-VIII, V cranial nerves, and superior cerebral artery (SCA) were identified for the retrosigmoid approach.

At the end of the workshop, quality and usability-grade surveys were filled out by participants. The second questionnaire was a modified version of the System Usability Scale (9). Each answer was graded by a 5-point scale (Likert score) (10). Incomplete questionnaires were excluded, and a total of 28 forms were considered for the assessment.

## Results

**Figure 4:**
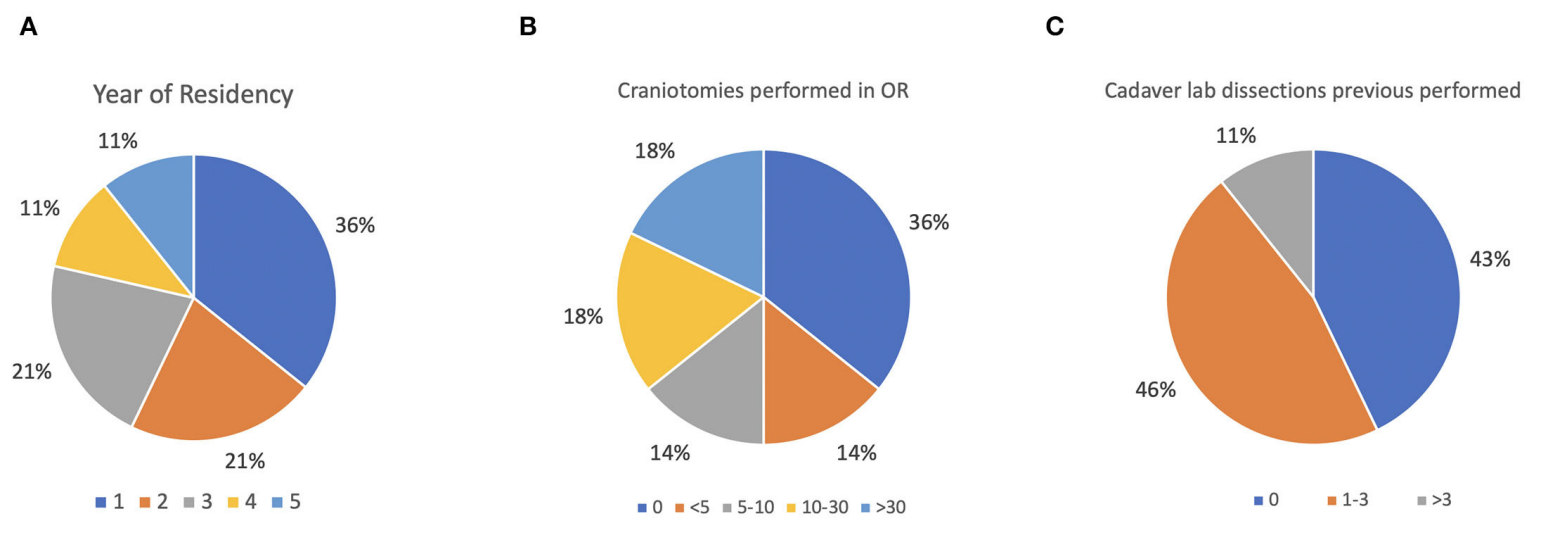
**(A–C)** Baseline assessment of the audience. **(A)** Stratification by year of neurosurgery residents; **(B)** assessment of performed craniotomies; **(C)** assessment of attended cadaver labs.

Descriptive results regarding stratifications by year of neurosurgery residents who participated in the surveys are reported in Figure 4A. In addition, the number of craniotomies and the number of previous cadaveric labs performed were assessed to define the experience level of the audience (Figures 4B,C). The results of the survey about teaching effectiveness and quality model are summarized in Table 1. More than 89% (agree and strongly agree) of the residents assessed that the application and the AR simulator were very helpful in improving the orientation skills during neurosurgical approaches. Indeed, 89.3% of the participants found brain and skull anatomy highly realistic during their tasks. Moreover, workshop exercises were considered useful in increasing the competency and technical skills required in operating room, by 85.8 and 84.7% of residents, respectively.

**Table 1:** Survey results about teaching effectiveness and quality of the model.

**Survey #1 item**	**Strongly disagree (1)**	**Disagree (2)**	**Neutral (3)**	**Agree (4)**	**Strongly agree (5)**
The Application and the AR simulator help to develop the orientation skills needed during neurosurgical approach	0.0	3.6	7.1	28.6	60.7
The BrainBox had appropriate surface anatomy	0.0	0.0	10.7	39.3	50.0
Neurovascular structures and skull base anatomy were realistic and appropriately detailed for surgical orientation	0.0	0.0	3.6	64.3	32.1
The tactile feedback and response on manipulation was realistic	0.0	3.7	44.4	37.0	14.8
Skills to handle the craniotomies and dissection instruments were representative of those required to perform the real procedure	0.0	0.0	15.4	38.5	46.2
The drilling experience is similar to the real skull	0.0	3.6	7.1	46.4	42.9
Dural opening and suturing was realistic	3.7	14.8	40.7	22.2	18.5
Using this model helps to increase competency when applied to neurosurgical training	0.0	0.0	14.3	17.9	67.9
I feel more confident using neurosurgical instruments after training with this model	0.0	0.0	21.4	35.7	42.9
Using this model can facilitate the process of using the surgical microscope	0.0	0.0	7.1	25.0	67.9
The study of the surgical approach and surgical anatomy in a virtual way (App) passing through augmented reality and then the BrainBox is an effective method of learning	0.0	0.0	3.6	39.3	57.1
This model of training should be part of a standard curriculum	0.0	0.0	14.3	25.0	60.7

On the other side, tactile feedback of the brain tissue and dura mater consistency were found as relative weaknesses of anatomical models with a mean score of 3 points (neutral). In particular, the most critical issue found was the lower elasticity and higher hardness and tension of the anatomical model compared to normal parenchyma.

Interestingly, when the residents were asked, 85.7% agreed with the possibility to include this kind of training into a standard surgical training for neurosurgeons (4 and 5 scores).

Data collected with the second survey confirmed that the anatomical model and its application were intuitive, well integrated, and easy to use. The results of grade of usability questionnaire about anatomy models are summarized in Table 2.

**Table 2:** Evaluation of the anatomical model through the System Usability Scale.

**Survey #2 item**	**Strongly disagree (1)**	**Disagree (2)**	**Neutral (3)**	**Agree (4)**	**Strongly agree (5)**
I think that I would like to use UpSurgeOn box frequently	0.0	0.0	14.3	42.9	42.9
I found UpSurgeOn box unnecessarily complex	35.7	50.0	7.1	7.1	0.0
I thought UpSurgeOn Box was easy to use	0.0	0.0	14.3	28.6	57.1
I think that I would need the support of a technical person to be able to use UpSurgeOn box	35.7	21.4	35.7	0.0	7.1
I found the various functions in UpSurgeOn box were well integrated	0.0	0.0	10.7	46.4	42.9
I found consistency between the functions of the UpSurgeOn box	0.0	0.0	0.0	28.6	71.4
I found the UpSurgeOn box very intuitive to use	0.0	0.0	14.3	32.1	53.6
I would imagine that most people would learn to use UpSuregOn box very quickly	0.0	0.0	0.0	14.3	85.7
I felt very confident using UpSurgeOn box	0.0	0.0	14.3	35.7	50.0
I needed to learn few things before I could get going with UpSurgeOn box	0.0	0.0	0.0	28.6	71.4

Moreover, although the aim of the study was not a quantitative analysis, data regarding length of exercises and performance qualitative evaluation are reported in Tables 3, 4. Results showed that residents attending the last 2 years of residency performed the various skills quicker and with higher quality than younger residents. However, no significant comparisons or analysis could be made on these data because of the heterogeneity of surgical approach among different groups of residents.

**Table 3:** Descriptive results regarding time, procedures, and quality of exercises.

**Residency Y**	**Approach**	**Time (Min:Sec)**	**Quality (1–5)**
**Craniotomy**			
1	Retrosigmoid	14:43	2
1	Retrosigmoid	13:10	2
1	Subtemporal	12:20	3
1	Pterional	16:45	3
2	Pterional	11:12	2
2	Subtemporal	15:10	3
2	Pterional	10:40	3
2	Subtemporal	08:20	4
2	Pterional	09:50	4
3	Pterional	08:38	4
3	Pterional	07:12	3
3	Retrosigmoid	06:24	4
4	Subtemporal	09:30	4
5	Retrosigmoid	05:34	5
5	Pterional	03:10	4
**Dural opening and suspension**			
1	Retrosigmoid	15:30	2
1	Retrosigmoid	12:45	1
1	Subtemporal	19:35	1
1	Pterional	23:45	2
2	Pterional	18:40	2
2	Subtemporal	14:20	3
2	Subtemporal	16:45	2
2	Pterional	17:10	3
2	Pterional	13:00	3
3	Pterional	09:20	3
3	Pterional	06:40	2
3	Retrosigmoid	05:50	4
4	Subtemporal	06:35	4
5	Retrosigmoid	04:00	4
5	Pterional	04:30	5
**Microscopic target**			
1	Retrosigmoid	09:40	1
1	Subtemporal	07:20	1
1	Retrosigmoid	08:30	2
1	Pterional	06:40	2
2	Pterional	11:10	2
2	Pterional	09:10	3
2	Subtemporal	06:10	2
2	Subtemporal	05:40	2
2	Pterional	08:30	3
3	Pterional	08:10	3
3	Retrosigmoid	06:05	4
3	Pterional	05:25	4
4	Subtemporal	02:40	4
5	Retrosigmoid	02:50	5
5	Pterional	02:20	4
**Dural closure**			
1	Pterional	16:50	1
1	Subtemporal	13:15	3
1	Retrosigmoid	15:10	2
1	Retrosigmoid	15:40	3
2	Subtemporal	10:35	3
2	Pterional	11:35	4
2	Pterional	12:10	3
2	Pterional	11:25	3
2	Subtemporal	10:40	2
3	Pterional	09:10	5
3	Retrosigmoid	07:50	4
3	Pterional	10:15	3
4	Subtemporal	05:20	5
5	Retrosigmoid	04:20	5
5	Pterional	05:40	5

**Table 4:** Descriptive results stratified by year of residency.

**Resident Y**	**Average time**	**Quality**
**Craniotomy**
1	14:14	2.50
2	11:02	3.20
3	07:24	3.67
4	09:30	4.00
5	04:22	4.50
**Dural opening**
1	23:53	1.5
2	15:59	2.6
3	07:16	3
4	06:35	4
5	04:15	4.5
**Microscopic target**
1	08:02	1,5
2	08:08	2,4
3	06:33	3,67
4	02:40	4
5	02:35	4,5
**Dural closure**
1	15:13	2.25
2	11:17	3
3	09:05	4
4	05:20	5
5	05:00	5

## Discussion

Neurosurgery residency is characterized by high levels of competence and an intense hands-on experience. Due to monetary restrictions, infrastructure conditions, and recent work time restrictions (11), it is hard for a resident to reach an adequate operative case volume over the education program (3). To counter these drawbacks in neurosurgical training, cadaveric specimen (12) and animal model courses (13) are some of the common alternatives for the improvement of surgical skills in neurosurgery, even if their high maintenance costs and ethical issues represent some of the principal limitations to date (14). Some abroad experiences or post-graduate surgical courses are still valid options, but recently, after the COVID-19 pandemic, international exchange programs have been dramatically reduced (6, 7). Also, learning new surgical skills is different from perfecting them, which implies that they have to be repeated with constancy over time (15). The neurosurgical learning curve is still too long and dominated by conventional mentor–apprentice relationships (16).

In this scenario, neurosurgical simulators are becoming increasingly important. Among the modern surgical training solutions, interest in VR or AR and 3D models has been growing (16, 17). Our experience with hybrid AR and 3D-printed neurosurgical simulator showed that the combination of such learning methods could lead to interesting results. Specifically, hybrid AR represents a helpful tool to guide young neurosurgeons from notional knowledge to practical experience. Indeed, after an anatomical revise, residents can focus on the correct patient positioning and go over the chosen approach step-by-step by interacting with the model through AR application. In the second phase of the simulation, high detailed 3D-printed neurosurgical models allow trainees to obtain immediate feedback of the previous theoretical topics.

Winkler-Schwartz et al. (18) have already described 17 students' and residents' training experience with a VR simulator. Their results suggest the possibility to categorize participants' technical abilities and use this tool to develop and maintain psychomotor skills. Licci et al. (2) developed a synthetic simulator based on patient-specific computed tomography (CT) data set, and different realistic skull models were produced by a 3D printer, including vascular structures and some soft tissue portions mimicking ventricle tumors. Neurosurgical trainees were invited to a neuroendoscopic workshop and qualitatively assessed afterwards. They found that this training empowered the development of specific surgical skills.

Joseph et al. (19) developed and used a physical simulator that was able to reproduce the experience of clipping intracranial aneurysms based on 3D-printed models of skull, brain, and arteries. They judged this simulator as a reliable and useful tool for neurosurgical training.

Chawla et al. recently reported a systematic review addressing 4 major neurosurgical skills using various modalities of training, assessed by face, content, and construct validity. An increased use of simulation models in neurosurgical training was found. Currently, synthetic models have been found to be the most convenient and practical, especially during the pandemic breakdown, but VR models are found promising due to the visual realism and improved haptic feedback technology (20).

The topic of burnout among neurosurgery residents is widely covered in scientific literature (21, 22). Training outside the operating room provides the possibility of practical education based on constructive criticism in a stress-free field. Surgical simulation allows the residents to perform a constant self-assessment of their growth from a technical and cognitive point of view. Personal growth and intellectual reward could be a real solution for the burnout issue. Furthermore, the repetitive use of a 3D model associated with AR would allow to standardize the surgical act in the different years of residency. More than 85% of residents agree or strongly agree (Table 1) to make this kind of simulation a part of the standard curriculum in neurosurgery. Our next target will be to create a surgical portfolio based on hybrid simulation to complete before joining the procedure in the operating room.

The 3D anatomic model allows to recreate even complex pathological conditions such as tumors and cerebral aneurysm. Indeed, senior residents were asked to perform an extra trial: clipping exercise on few 3D models enhanced with saccular aneurysm of middle cerebral artery (MCA), anterior communicating artery (ACoA) and posterior communicating artery (PCoA). This is certainly an important strength of the simulator that human or animal cadaveric specimens cannot provide. It is conceivable that it will be possible to perform the same procedure several times on the same patient in a simulated way before arriving at the day of the planned surgery.

On the other side, the texture and tactile feedback of the brain tissue and dura mater can still be improved. These characteristics were assessed with a mediocre scores in about 40% of the responses (3 out of 5 points). Furthermore, the arachnoid and the cisterns, which constitute some of the anatomical references for the surgery of the skull base, are not represented. These are the current limits of the tool.

The hybrid simulation with AR and 3D model represents a constant, modular, and repeatable training tool, while the surgeon will be the only variable. This new way of learning could change the old rules of knowledge transmission in neurosurgery centered on the mentor–apprentice relationship.

### Limitations

The main limitation of this study is its purely qualitative nature and lack of quantitative analysis. However, as reported in the method section, this study was a preliminary experience with this new hybrid simulation, and the purpose of the study was primarily to assess the usability and liking of the tool. Therefore, despite its qualitative nature, the encouraging results that emerged from the survey could be considered a driving force for further quantitative studies aimed at analyzing the actual possibility of improving the learning curve of residents outside the operating room, using a reproducible and less expensive tool. To this end, the authors are developing a standardized 1-year surgical training program, tailored to the needs of different residency years, in which the residents' skills are assessed with quantitative scales in order to evaluate their growth curve.

## Conclusion

The hybrid AR and 3D-printed neurosurgical simulator could be a valid tool for neurosurgical training, which is capable of enhancing the technical skills and competence of the residents. In addition, it could be easy to imagine how patient safety would increase and healthcare costs would be reduced, even if more studies are needed to investigate these aspects. The integration of simulators for training in neurosurgery as preparatory steps for the operating room should be recommended and further investigated given their huge potential.

## Data Availability Statement

The raw data supporting the conclusions of this article will be made available by the authors, without undue reservation.

## Ethics Statement

Written informed consent was obtained from the individual(s) for the publication of any potentially identifiable images or data included in this article.

## Author Contributions

SP: writing draft. FC: editing and writing. FN: conceptualization and reviewing. GS: conceptualization and supervision. MM: conceptualization and data collection. GD: editing and data collection. AL: data collection. ML and DG: supervision and reviewing. All authors contributed to the article and approved the submitted version.

## Conflict of Interest

FN is Founder and CEO of UpSurgeOn. GS is Co-founder of UpSurgeOn. MM is Lead Bioengineer at UpSurgeOn. The remaining authors declare that the research was conducted in the absence of any commercial or financial relationships that could be construed as a potential conflict of interest. The handling editor declared a past co-authorship with several of the authors FC and DG.

## Publisher's Note

All claims expressed in this article are solely those of the authors and do not necessarily represent those of their affiliated organizations, or those of the publisher, the editors and the reviewers. Any product that may be evaluated in this article, or claim that may be made by its manufacturer, is not guaranteed or endorsed by the publisher.
